# Ethylene-releasing plant growth regulators promote ripening initiation by stimulating sugar, acid and anthocyanin metabolism in blueberry (*Vaccinium ashei*)

**DOI:** 10.1186/s12870-025-06799-x

**Published:** 2025-06-05

**Authors:** Tej P. Acharya, Savithri U. Nambeesan

**Affiliations:** 1 Department of Horticulture, University of Georgia, 1111 Miller Plant Sciences Building, Athens, GA 30602 USA; 2 U.S. Department of Agriculture, Agriculture Research Service, U.S. Horticultural Research Laboratory, Citrus and Other Subtropical Products Research Unit, 2001 South Rock Road, Fort Pierce, 34945 FL USA; 3 Indian River Research and Education Center, University of Florida, 2199 S Rock Rd, Fort Pierce, FL 34945 USA

**Keywords:** ACC, Anthocyanin, Ethephon, Fruit quality, Organic acid, Sugar

## Abstract

**Background:**

Fruit ripening is a coordinated process that leads to an increase in sugars, decrease in acids and accumulation of pigments. Blueberry fruit exhibit an atypical climacteric ripening behavior. These fruit display an increase in respiration and ethylene production during ripening, however ethylene synthesis is developmentally regulated. In this study, the effect of ethylene on blueberry fruit ripening was investigated *via* preharvest applications of ethylene-releasing plant growth regulators (PGRs), ethephon and 1-aminocyclopropane 1-carboxylic acid (ACC), in one southern highbush cultivar, Miss Lilly in 2019, and two rabbiteye cultivars, Premier and Powderblue in 2019 and 2020. Further, the effects of these two PGRs on fruit metabolism during ripening in the two rabbiteye cultivars, and postharvest fruit quality in all three cultivars were evaluated.

**Results:**

Both PGRs increased ethylene evolution within 1–3 days after treatment (DAT). Ethephon and ACC applications increased the rate of ripening within 5 DAT in all cultivars, and increased ripe (blue) fruit by up to 35% and 29%, respectively between 7 to 10 DAT compared to the control. Metabolite analysis revealed that PGR treatments resulted in an immediate, but transient increase in sucrose, glucose and fructose, in ‘Premier’ at 3 DAT. Malate decreased at 3 DAT in response to both PGR treatments in ‘Premier’, and at 5 DAT in ethephon treatment in both cultivars. A rapid increase in the concentration of multiple anthocyanins was noted at 3 DAT in response to both PGRs in ‘Premier’ and ‘Powderblue’. Gene expression analysis revealed an increase in transcript abundance of *VACUOLAR INVERTASE* (*vINV*) and multiple anthocyanin biosynthesis genes between 1 and 3 DAT after PGR treatments in both cultivars, supporting the metabolite changes. However, the alteration in fruit metabolite concentrations were not sustained, and similar in PGR-treated fruit compared to the control in ripe fruit harvested at 10 DAT. Postharvest fruit quality attributes, such as firmness, total soluble solids, titratable acidity, and visual quality, were not consistently affected by the PGR applications compared to control treatments across all cultivars. A decrease in fruit weight was noted, although not consistently, in response to PGR treatments.

**Conclusions:**

Overall, this study demonstrates that ethylene plays a crucial role in promoting ripening *via* rapid and transient stimulation of sugar, acid and anthocyanin metabolism. The promotion of fruit ripening by ethylene-releasing PGRs can lead to minimal but inconsistent changes in fruit quality attributes during postharvest storage.

**Supplementary Information:**

The online version contains supplementary material available at 10.1186/s12870-025-06799-x.

## Background

Blueberry fruit growth and development can be divided into three stages. Fruit growth in Stage 1 is initiated after fruit set and is mainly facilitated by cell division. Stage II is a lag phase with no substantial increase in fruit size, however during this period seed maturation occurs. Subsequently, growth resumes during Stage III mainly through cell expansion, and this stage is followed by ripening [1–3]. Fruit ripening is an integration of multiple physiological processes leading to an increase in sugars, decrease in acidity, accumulation of pigments and fruit softening [4, 5]. All fruits display the ripening syndrome. However, based on the underlying ripening physiology a binary classification has been adopted. Fruits such as banana, tomato, and apple display an increase in respiration and ethylene production during ripening and are classified as displaying climacteric ripening behavior [4, 6]. In these fruits, ethylene production is under autocatalytic regulation and this hormone is important for triggering ripening-related physiological changes. In contrast, non-climacteric fruits such as citrus, strawberry, and grape do not display a rise in ethylene and a respiratory peak during ripening. In these fruits the role of ethylene in facilitating ripening is not completely understood. Nevertheless, some studies have shown an increase in ethylene production during strawberry and grape ripening, although at considerably lower levels compared with climacteric fruit [7, 8]. More recently it has been proposed that fruits may fall along a spectrum of climacteric/non-climacteric behavior. In this regard, blueberry fruit exhibit atypical climacteric ripening physiology with a rise in respiration and ethylene. The regulation of ethylene appears to be under developmental control rather than being autocatalytic [9]. Previously, our work showed that upon application of ethephon, an ethylene-releasing plant growth regulator, ripening is accelerated [10]. Ethylene accelerates ripening by promoting the developmental transition into ripening by downregulating photosynthesis-related genes [11]. However, the role of ethylene in regulating fruit metabolism during blueberry ripening is not well understood.

During ripening, fruits undergo extensive changes in their metabolite composition and differences in associated genes. Some of the metabolic changes that occur during blueberry ripening are summarized below. In blueberry, a continuous increase in the concentration of the three main sugars, glucose (Glc), fructose (Fru) and sucrose (Suc) is noted during fruit development and ripening [12]. Sucrose, which is translocated into the fruit from leaves can be hydrolyzed by two enzymes: cytosolic sucrose synthase (SuSy) into UDP-Glc and Fru, and by invertases (INV) into Glc and Fru [13, 14]. There are three types of INV: cell wall INV (cwINV) involved in breakdown of Suc that is apoplastically unloaded into the fruit, and cytosolic (cINV) and vacuolar invertases (vINV) that hydrolyze Suc in their respective cellular compartments [14, 15]. Of these, the transcript abundance of *vINV* is dramatically higher during blueberry fruit development compared to other sucrose catabolic enzymes, suggesting its importance in sugar metabolism [12]. The hexose sugars generated from Suc catabolism can be channeled towards primary metabolic pathways such as glycolysis, the synthesis of organic acids and TCA cycle, the mitochondrial electron transport chain (mETC) for generation of energy, providing carbon skeletons for secondary metabolites, and cell wall biosynthesis. In blueberry fruit, the major primary organic acids are malate and citrate [12, 16]. The glycolytic intermediate phophoenolpyruvate (PEP) generates malate in a two-step process, using PEP carboxylase (PEPC) to generate oxaloacetate (OAA) which is subsequently converted to malate *via* malate dehydrogenase (MDH) activity [17, 18]. The pattern of transcript abundance of these genes suggests that during blueberry fruit development leading up to ripening there is an accumulation of malate. During ripening, malate appears to be converted to OAA and then *via* PEP carboxykinase (PEPCK) to PEP [12]. PEP is an important substrate for the generation of secondary acids such as quinate and shikimate [19, 20]. The concentration of quinate itself is high in blueberry fruit during mid-development and decreases during ripening [12]. Quinate catabolism can potentially contribute to shikimate biosynthesis. Shikimate can be used to generate secondary metabolites including anthocyanins which are abundant in ripe blueberry fruit [12, 21]. From a human nutrition standpoint, anthocyanins offer numerous health benefits such a decreased risk of cardiovascular diseases and diabetes, and enhanced cognitive performance [22].

The role of ethylene in influencing metabolic shifts during ripening in blueberry is not understood. As the ripening physiology of blueberry is distinct due to its atypical climacteric behavior, it presents opportunities for deeper insights into the role of ethylene in stimulating fruit metabolism in comparison to climacteric and non-climacteric fruits. Therefore, the objective of this study was to determine the role of ethylene in blueberry fruit metabolism during ripening. This was achieved through the application of ethylene-releasing PGRs, ethephon and 1-aminocyclopropane 1-carboxylic acid (ACC). The effect of increased ethylene on sugar, acid and anthocyanin metabolism and related gene expression was investigated during the course of ripening. Further the effect of preharvest applications of ethylene-releasing PGRs and their effect on postharvest fruit quality was evaluated.

## Results

### Effects of ethephon and ACC treatments on rate of ripening

**Fig. 1:**
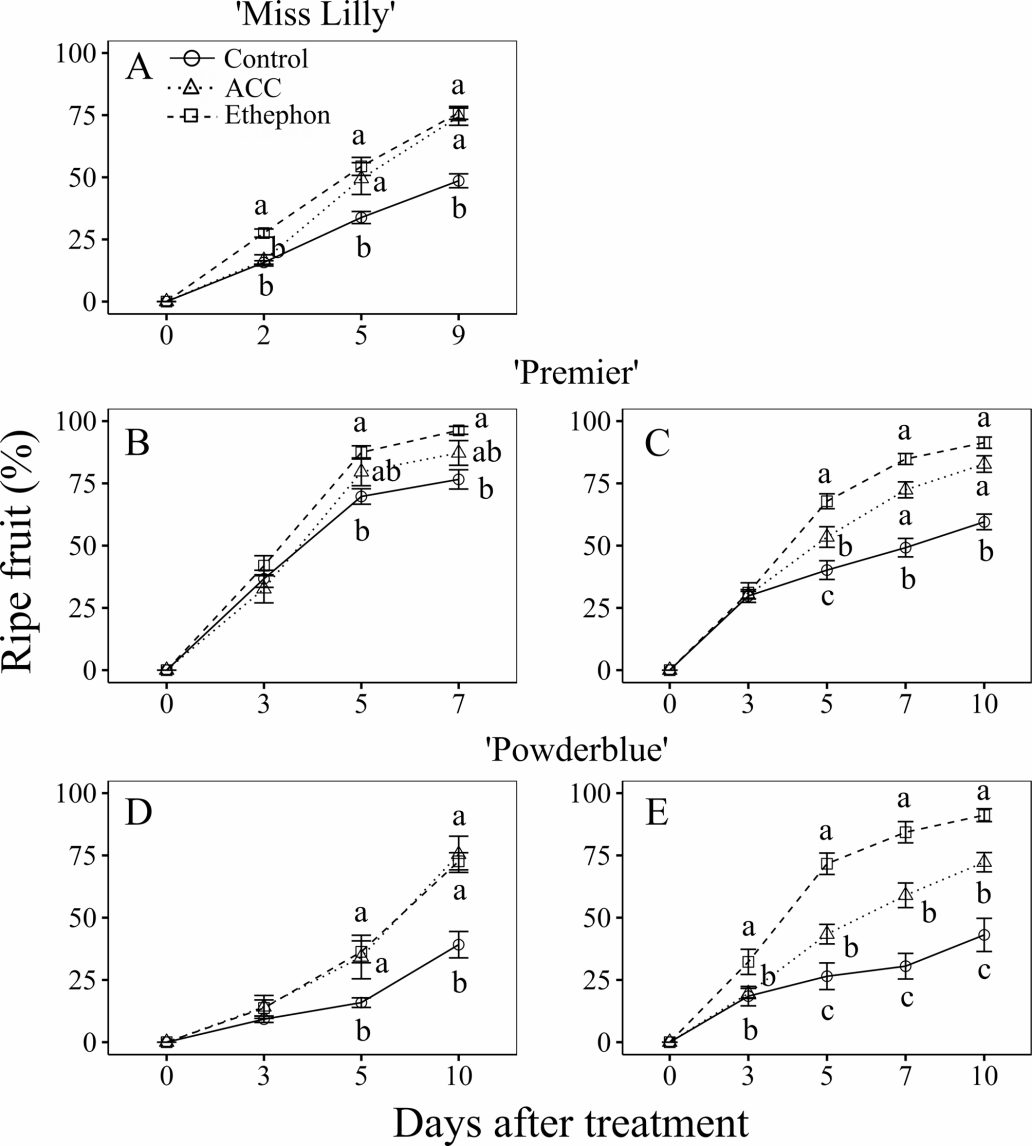
Effects of ethephon and 1-aminocyclopropane 1-carboxylic acid (ACC) treatments on rate of ripening in blueberry. Percentage of ripe fruit in control and in response to ethephon and ACC treatments in three cultivars, Miss Lilly (**A**), Premier (**B**, **C**) and Powderblue (**D**, **E**) in 2019 (**A**, **B**, **D**) and 2020 (**C**, **E**). Mean ± S.E. (*n* = 4) are presented. Statistical analyses were performed using ANOVA followed by Fischer’s Least Significant Difference (LSD; *α* = 0.05). The same letter above the symbols indicates no statistically significant differences across treatments within a given day after treatment

Overall, an increase in the rate of ripening in response to the PGR treatments was noted in all cultivars and in both years of the study (Fig. 1). In 2019, in ‘Miss Lilly’, ethephon increased ripe fruit percentage by 75.3%, 60.9%, and 56.2% compared to the control at 2, 5, and 9 DAT, respectively (Fig. 1A). Similarly, in ‘Miss Lilly’, ACC increased ripe fruit percentage by 46.4% and 53.1% at 5 and 9 DAT, respectively. In 2019, in ‘Premier’, ethephon increased ripe fruit percentage by 25.4% and 25.7% compared to the control at 5 and 7 DAT, respectively (Fig. 1B), and in 2020 by 68.8%, 72.4% and 53.4% compared to control at 5, 7, and 10 DAT, respectively (Fig. 1C). In 2019, in ‘Premier’, ACC displayed an increasing trend (not significant) in percent ripe fruit, whereas in 2020, ACC treatments increased the percentage of ripe fruit by 33.1%, 47.2%, and 39.0% compared to the control at 5, 7, and 10 DAT, respectively (Fig. 1C). Similar results on rate of ripening after PGR treatments were noted in ‘Powderblue’. In ‘Powderblue’, in 2019, ethephon and ACC increased the percentage of ripe fruit compared to the control at 10 DAT by 85.5% and 92.6%, respectively (Fig. 1D), and in 2020 by 111.6% and 67.8%, respectively (Fig. 1E).

In general, the decrease in percent green fruit after ethephon and ACC treatments complemented the increase in ripe fruit in the three cultivars during both years (Fig. S1A-E). Only for Premier in 2019, where the data for proportions of green and pink fruit were combined, ethephon treatments decreased their proportion compared to the control treatment (Fig. S1A). Further, ethephon and ACC transiently increased the proportion of pink fruit at 5 DAT in ‘Miss Lilly’. Similarly, ACC increased the proportion of pink fruit at 5 and 7 DAT in ‘Powderblue’ and ‘Premier’, respectively in 2020. Ethephon increased the proportion of pink fruit at 3 and 5 DAT in ‘Powderblue’ in both years (Fig. S1F-I).

### Effects of ethephon and ACC treatments on ethylene and CO_2_ production

**Fig. 2:**
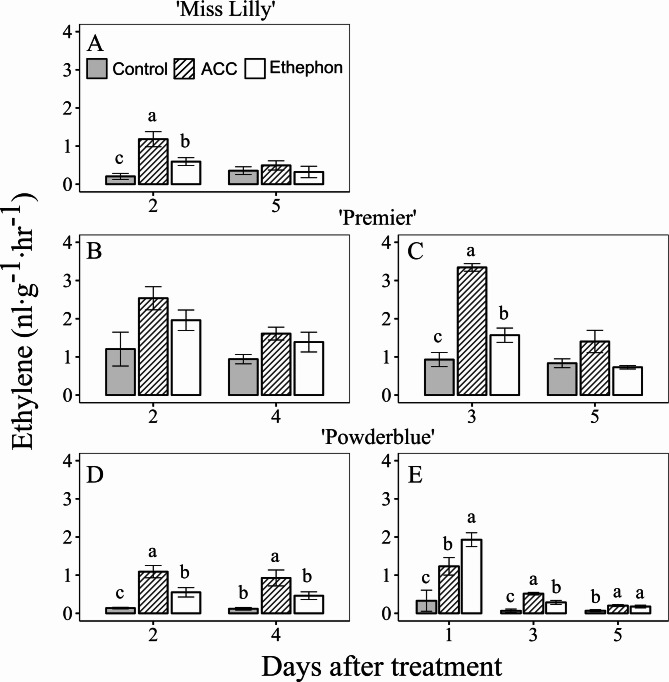
Ethylene concentration in blueberry fruit in response to ethephon and 1-aminocyclopropane 1-carboxylic acid (ACC) treatments. Ethylene concentrations in control and in response to ethephon and ACC treatments were determined in three cultivars, Miss Lilly (**A**), Premier (**B**, **C**) and Powderblue (**D**, **E**) in 2019 (**A**, **B**, **D**) and 2020 (**C**, **E**). A pool of fruit was randomly collected from tagged branches for each time-point after treatments. Mean ± S.E. (*n* = 4) are presented. Statistical analyses were performed using ANOVA followed by Fischer’s Least Significant Difference (LSD; *α* = 0.05). The same letter above the bars indicates no statistically significant differences across treatments within a given day after treatment

**Fig. 3:**
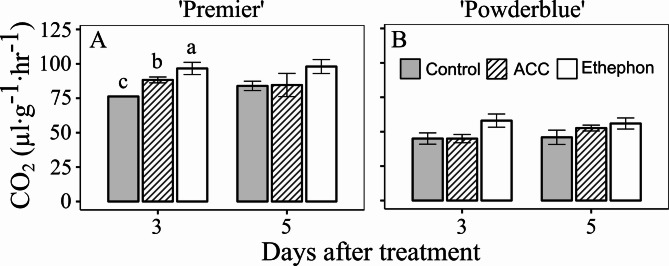
Respiration rate in blueberry fruit after ethephon and 1-aminocyclopropane 1-carboxylic acid (ACC) treatments. Blueberry fruit carbon dioxide evolution rate in control and in response to ethephon and ACC treatments in two cultivars, Premier (**A**) and Powderblue (**B**) in 2020. A pool of fruit was randomly collected from tagged branches for each time-point after treatments. Mean ± S.E. (*n* = 4) are presented. Statistical analyses were performed using ANOVA followed by Fischer’s Least Significant Difference (LSD; *α* = 0.05). The same letter above the bars indicates no statistically significant differences across treatments within a given day after treatment

In general, ethephon and ACC treatments increased ethylene production compared with the control (Fig. 2). In 2019, ethylene production in the control treatment at 2 DAT was 0.21, 1.20, and 0.14 nL∙g^− 1^∙hr^− 1^ in ‘Miss Lilly’, ‘Premier’, and ‘Powderblue’, respectively. In 2020, it was 0.93 and 0.07 nL∙g^− 1^∙hr^− 1^ at 3 DAT in ‘Premier’ and ‘Powderblue’, respectively. Ethephon and ACC increased ethylene evolution by 2.9- and 5.7-fold at 2 DAT in ‘Miss Lilly’ in 2019 and by 1.7- and 3.6-fold at 3 DAT in ‘Premier’ in 2020, respectively compared to the control (Fig. 2A, C). In 2019, in ‘Premier’, the two PGRs displayed a trend of increased ethylene production (not significant) at 2 DAT (Fig. 2B). Ethephon also increased ethylene production compared to the control by 4-fold at 2 DAT in ‘Powderblue’ in 2019 and by 5.8-, 4.2-, and 2.5-fold at 1, 3, and 5 DAT, respectively, in ‘Powderblue’ in 2020. Similarly, ACC increased ethylene production compared to the control by 7.9- and 7.6-fold at 2 and 4 DAT in ‘Powderblue’ in 2019 and by 3.7-, 7.5-, and 2.9–fold in ‘Powderblue’ in 2020, respectively (Fig. 2D, E). Compared to the control, both ethephon and ACC increased CO_2_ production by 1.3- and 1.2-fold at 3 DAT in ‘Premier’ (Fig. 3A), but this response was not detected in ‘Powderblue’ in 2020 (Fig. 3B).

### Effects of ethephon and ACC treatments on sugar metabolism

**Fig. 4:**
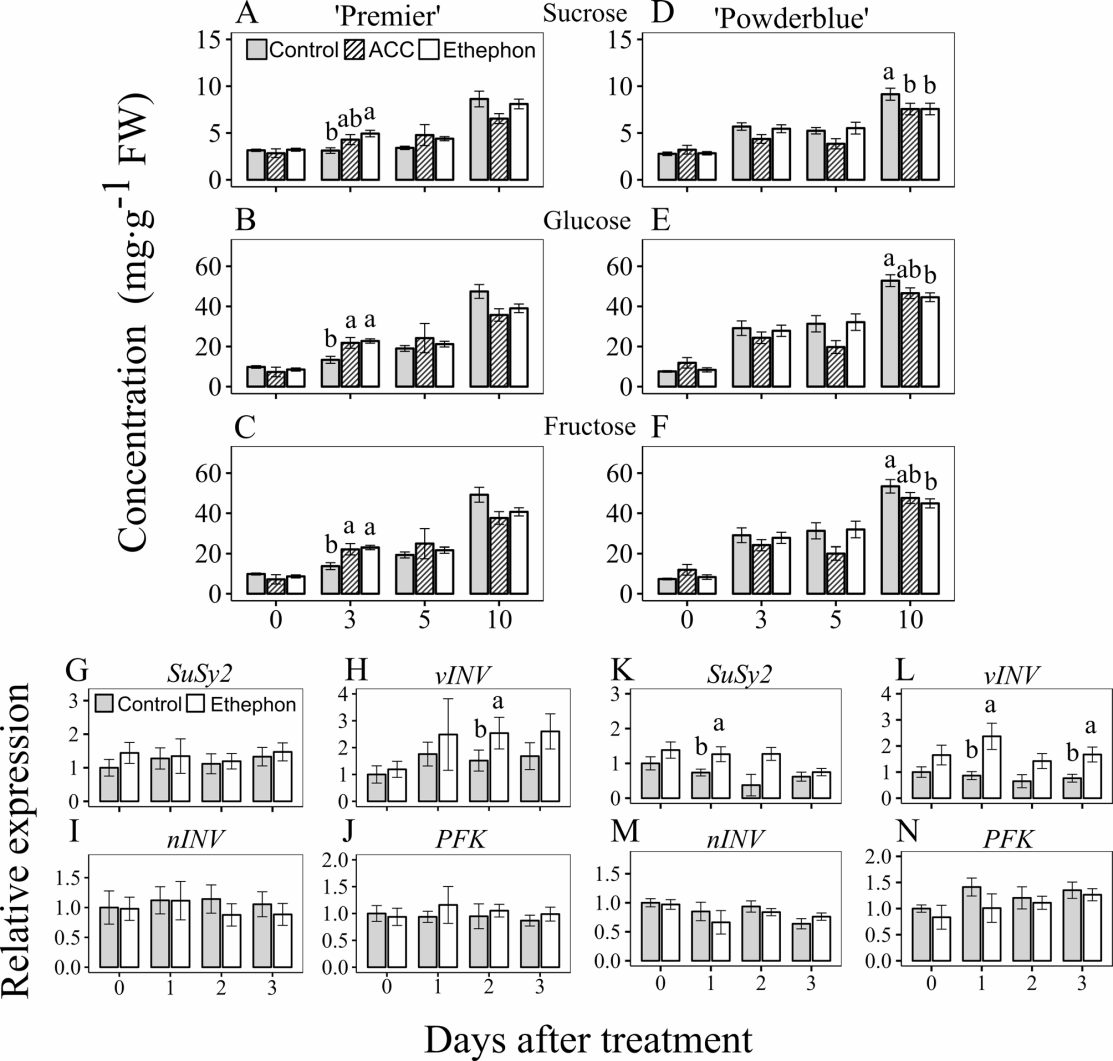
Effects of ethephon and 1-aminocyclopropane 1-carboxylic acid (ACC) treatments on sugar metabolism and related gene expression in blueberry fruit. Concentration of sucrose (**A**, **D**), glucose (**B**, **E**), and fructose (**C**, **F**) in control fruit and after treatments with ethephon and ACC in ‘Premier’ (**A**-**C**) and ‘Powderblue’ (**D**-**F**) in 2020 at 0, 3, 5 and 10 days after treatment (DAT) were determined. Transcript abundance of *SUCROSE SYNTHASE 2* (*SuSy2*; **G**, **K**), *VACUOLAR INVERTASE* (*vINV*; **H**, **L**), *NEUTRAL INVERTASE* (*nINV*; **I**, **M**), and *PHOSPHOFRUCTOKINASE* (*PFK*; **J**, **N**) genes after ethephon and control treatments in ‘Premier’ (**G**-**J**) and ‘Powderblue’ (**K**-**N**) were determined in 2020 at 0, 1, 2 and 3 DAT. Transcript abundance data are presented in reference to the control fruit at 0 DAT. A pool of fruit was randomly collected from tagged branches for each time-point after treatment, except at 10 DAT where only ripe fruit were harvested. Mean ± S.E. (*n* = 4) are presented. Statistical analyses were performed using ANOVA followed by Fischer’s Least Significant Difference (LSD; *α* = 0.05). The same letter above the bars indicates no statistically significant differences across treatment(s) within a given day after treatment

Compared to the control, ethephon increased the concentration of Suc by 1.6-fold at 3 DAT and that of Glc and Fru by 1.7-fold in ‘Premier’ (Fig. 4A-C). Similarly, ACC compared to the control increased the concentration of Glc and Fru by 1.6-fold in ‘Premier’ at 3 DAT. However, no treatment effects on these metabolites were noted in ripe fruit at 10 DAT. The sugar-alcohol, myo-inositol, found at much lower concentrations than the major sugars, showed a slight increase at 10 DAT in ‘Premier’ (1.2-fold) following ethephon treatment (Fig. S2A). In ‘Powderblue’, ethephon and ACC treatments did not affect the concentration of major sugars and myo-inositol at 3 and 5 DAT. At 10 DAT, control fruit displayed 1.2-fold increase in concentrations of Suc, Glc, and Fru in comparison to ethephon-treated fruit (Fig. 4D-F; Fig. S2F).

The transcript abundance of sugar metabolism and early glycolysis-related enzymes in response to ethephon treatments was determined. The transcript abundance of *SuSy2* was not different in response to ethephon in ‘Premier’, however, in ‘Powderblue’, it increased by 1.7-fold at 1 DAT compared to the control (Fig. 4G, K). The transcript abundance of *vINV* increased by 1.7-fold at 2 DAT in ‘Premier’, and by 2.7- and 2.2-fold at 1 and 3 DAT, respectively in ‘Powderblue’ in ethephon treated fruit compared to the control (Fig. 4H, L). Ethephon did not influence the transcript abundance of *nINV* and *PHOSPHOFRUCTOKINASE* (*PFK*) in both cultivars (Fig. 4I, J, M, N).

### Effects of ethephon and ACC treatments on organic and amino acid metabolism

**Fig. 5:**
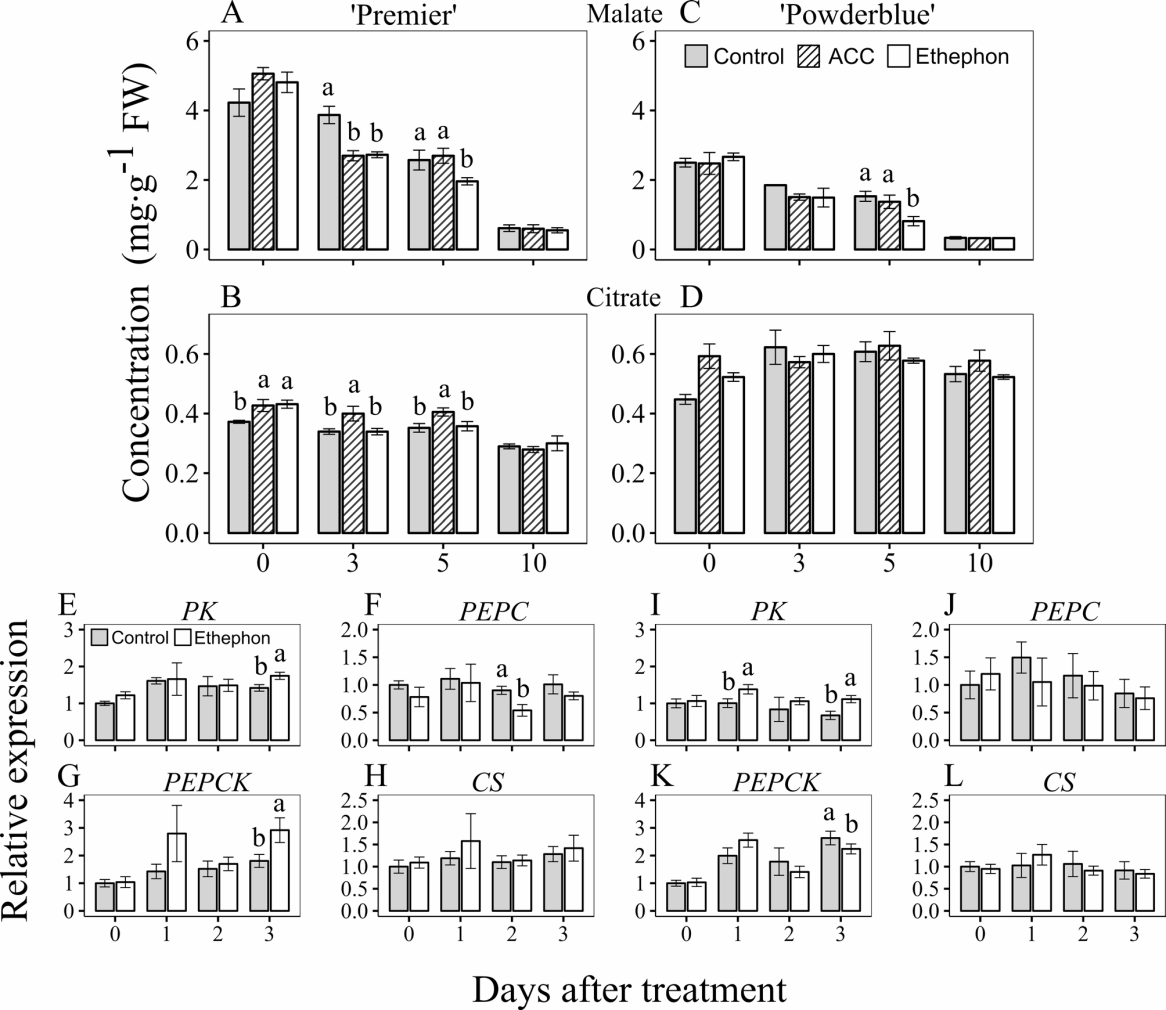
Effects of ethephon and 1-aminocyclopropane 1-carboxylic acid (ACC) treatments on acid metabolism and related gene expression in blueberry fruit. Concentrations of malate (**A**, **C**) and citrate (**B**, **D**) in control fruit and after treatment with ethephon and ACC in ‘Premier’ (**A**, **B**) and ‘Powderblue’ (**C**, **D**) were measured in 2020 at 0, 3, 5 and 10 days after treatment (DAT). Transcript abundance of *PYRUVATE KINASE* (*PK*; **E**, **I**), *PHOSPHOENOLPYRUVATE CARBOXYLASE* (*PEPC*; **F**, **J**), *PHOSPHOENOLPYRUVATE CARBOXYKINASE* (*PEPCK*; **G**, **K**), and *CITRATE SYNTHASE* (*CS*; **H**, **L**) were determined after ethephon and control treatments in ‘Premier’ (**E**-**H**) and ‘Powderblue’ (**I**-**L**) in 2020 at 0, 1, 2 and 3 DAT. Transcript abundance data are presented in reference to the control fruit at 0 DAT. A pool of fruit was randomly collected from tagged branches for each time-point after treatment except for 10 DAT, where only ripe fruit were harvested. Mean ± S.E. (*n* = 4) are presented. Statistical analysis was performed using ANOVA followed by Fischer’s Least Significant Difference (LSD; *α* = 0.05). The same letter above the bars indicates no statistically significant differences across treatment(s) within a given day after treatment

In comparison to the control, ethephon treatment decreased malate concentration by 1.4- and 1.3-fold at 3 and 5 DAT, respectively in ‘Premier’, and by 1.9-fold at 5 DAT in ‘Powderblue’ (Fig. 5A, C). Similarly, ACC decreased malate concentration by 1.4-fold at 3 DAT in ‘Premier’. There were no treatment effects in response to ethephon and ACC on malate concentration in ripe fruit at 10 DAT in both cultivars. Overall no effects of ethephon and ACC were noted on citrate and quinate concentration in both cultivars (Fig. 5B, D; Fig. S2B, G). Ethephon-treated fruit displayed an increase in concentration of shikimate compared to the control at 3 and 5 DAT by 1.5- and 1.8-fold, respectively, in ‘Premier’ (Fig. S2C). This effect was not observed with ACC treatment. In ‘Powderblue’, the concentration of shikimate was not different after treatments at any of the time-points evaluated, with the exception of 10 DAT where ethephon treatment resulted in slightly decreased concentration compared to the control (Fig. S2H). The concentration of amino acid, aspartate, did not change in response to the PGR treatments in both cultivars (Fig. S2D, I). Ethephon and ACC increased glutamate concentration by 2.4- and 2.2-fold, respectively at 5 DAT in ‘Premier’ but no change was observed in ‘Powderblue’ (Fig. S2E, J).

The transcript abundance of late glycolysis-related enzymes and acid metabolism were determined between ethephon and controls treatments at 0, 1, 2, and 3 DAT (Fig. 5E-L). Ethephon increased the transcript abundance of *PYRUVATE KINASE* (*PK*) by 1.2-fold at 3 DAT compared to the control in ‘Premier’, and by 1.4- and 1.7-fold at 1 and 3 DAT, respectively in ‘Powderblue’ (Fig. 5E, I). Ethephon decreased the transcript abundance of *PEPC* by 1.7-fold at 2 DAT in ‘Premier’, but no change was observed in ‘Powderblue’ (Fig. 5F, J). The effect of ethephon on *PEPCK* expression was inconsistent between cultivars. At 3 DAT, ethephon treatment increased the transcript abundance of *PEPCK* by 1.6-fold in ‘Premier’, whereas its expression decreased by 1.2-fold in ‘Powderblue’ (Fig. 5G, K). The transcript abundance of *CS* did not change in response to ethephon treatment in both cultivars (Fig. 5H, L).

### Effects of ethephon and ACC treatments on anthocyanin metabolism

**Fig. 6:**
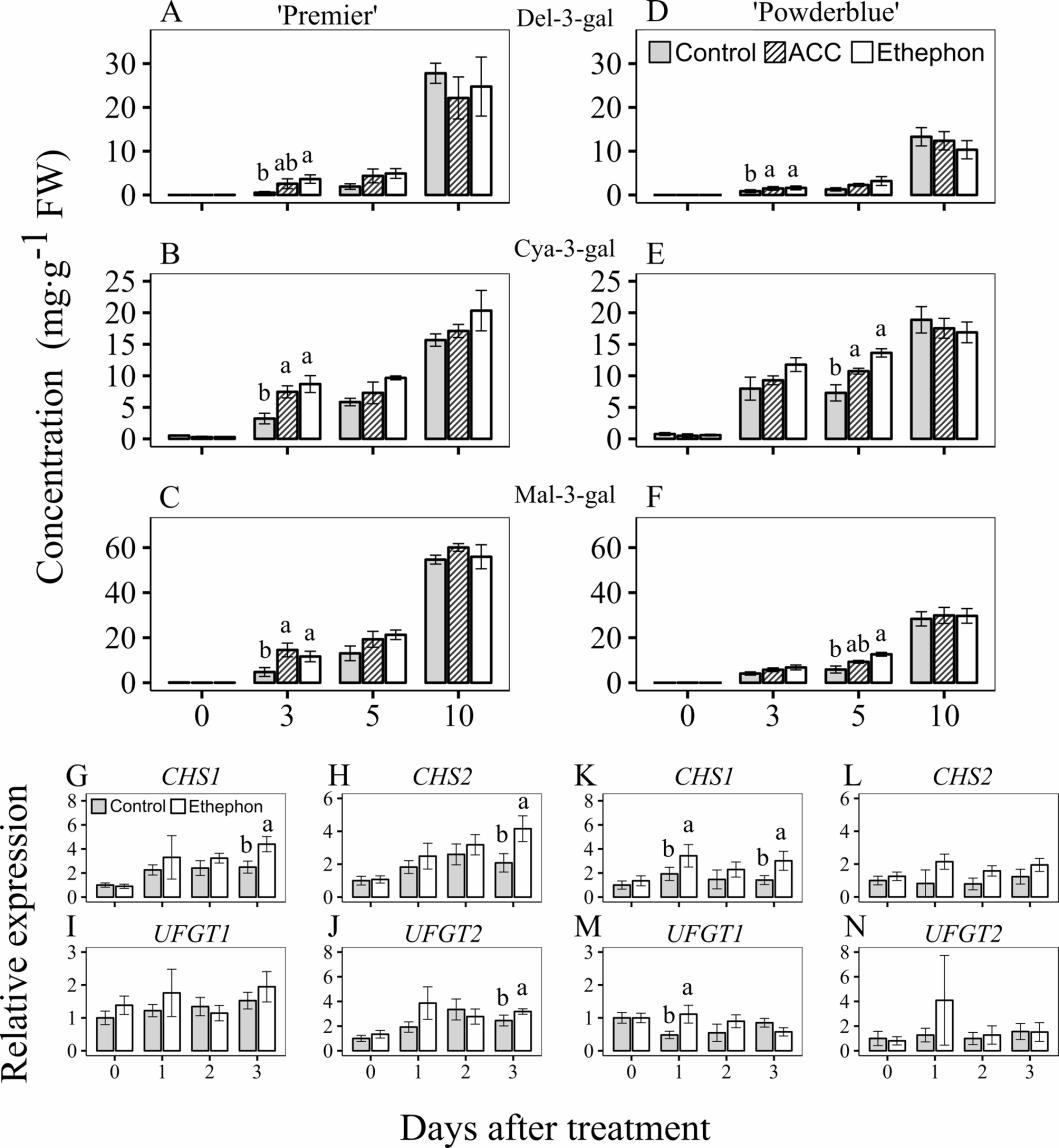
Effects of ethephon and 1-aminocyclopropane 1-carboxylic acid (ACC) treatments on anthocyanin metabolism and related gene expression in blueberry fruit. Concentrations of delphinidin 3-galactoside (Del-3-gal; **A**, **D**), cyanidin 3-galactoside (Cya-3-gal; **B**, **E**), and malvidin 3-galactoside (Mal-3-gal; **C**, **F**) in control fruit and after treatment with ethephon and ACC were determined in ‘Premier’ (**A**-**C**) and ‘Powderblue’ (**D**-**F**) in 2020 at 0, 3, 5 and 10 days after treatment (DAT). Transcript abundance of *CHALCONE SYNTHASE 1* (*CHS1*; **G**, **K**), *CHALCONE SYNTHASE 2* (*CHS2*; **H**, **L**), *ANTHOCYANIDIN 3-O-GLUCOSYLTRANSFERASE 1* (*UFGT1*; **I**, **M**), and *ANTHOCYANIDIN 3-O-GLUCOSYLTRANSFERASE 2* (*UFGT2*; **J**, **N**) in control fruit and after ethephon treatment in ‘Premier’ (**G**-**J**) and ‘Powderblue’ (**K**-**N**) were determined in 2020 at 0, 1, 2 and 3 DAT. Transcript abundance data are presented in reference to the control fruit at 0 DAT. A pool of fruit was randomly collected from tagged branches for all time-points after treatment except for 10 DAT, where only ripe fruit were harvested. Mean ± S.E. (*n* = 4) are presented. Statistical analyses were performed using ANOVA followed by Fischer’s Least Significant Difference (LSD; *α* = 0.05). The same letter above the bars indicates no statistically significant differences across treatment(s) within a given day after treatment

Totally 15 anthocyanins were detected including delphinidin 3-galactoside (Del 3-gal), delphinidin 3-glucoside (Del 3-glu), delphinidin 3-arabinoside (Del 3-ara), cyanidin 3-galactoside (Cya 3-gal), cyanidin 3-glucoside (Cya 3-glu), cyanidin 3-arabinoside + petunidin 3-galactoside (Cya 3-ara + Pet 3-gal), petunidin 3-glucoside (Pet 3-glu), petunidin 3-arabinoside (Pet 3-ara), peonidin 3-galactoside (Peo 3-gal), peonidin 3-glucoside (Peo 3-glu), peonidin 3-arabinoside (Peo 3-ara), malvidin 3-galactoside (Mal 3-gal), malvidin 3-glucoside (Mal 3-glu) and malvidin 3-arabinoside (Mal 3-ara) (Fig. 6; Table S1, S2). In ‘Premier’, treatment with ethephon and ACC, resulted in an average 3.6-fold increase in most anthocyanins particularly by 3 DAT, with the exception of Del 3-glu, Del 3-ara, and Pet 3-glu (Fig. 6A-C; Table S1). In ‘Powderblue’ all anthocyanins, except for Del 3-ara, Pet 3-glu, and Peo 3-gal, increased on an average by 2-fold after PGR treatments at 3 and/or 5 DAT (Fig. 6D-F; Table S2).

The transcript abundance of anthocyanin biosynthesis genes in response to ethephon treatment was determined (Fig. 6G-N). Ethephon treatment increased the transcript abundance of *CHALCONE SYNTHASE1* (*CHS1*) and *CHS2* by 1.8- and 2-fold, respectively at 3 DAT in ‘Premier’. In ‘Powderblue’ ethephon treatment increased *CHS1* expression by 1.8- and 2.1-fold at 1 and 3 DAT, respectively, however *CHS2* expression was similar compared to the control (Fig. 6G, H, K, L). Ethephon treatment increased the transcript abundance of *UDP-GLUCOSE: FLAVONOID 3-O-GLUCOSYLTRANSFERASE* (*UFGT1*) by 2.3-fold at 1 DAT only in ‘Powderblue’ and that of *UFGT2* by 1.3-fold at 3 DAT only in ‘Premier’ (Fig. 6I, J, M, N).

### Effects of PGR treatments on postharvest fruit quality attributes

Control and PGR treated ripe fruit harvested between 7 and 10 DAT were used to determine postharvest (PH) fruit quality (Tables 1 and 2). In general, the treatments did not affect fruit firmness consistently among cultivars and between years (Table 1). The exceptions were only in ‘Powderblue’, which included higher compression values at PH21 in 2020 in response to ethephon, and higher puncture values at PH21 in 2019 and PH3 in 2020, with either PGR treatments (Table 1).

**Table 1:** Effects of ethephon and 1-aminocyclopropane 1-carboxylic acid (ACC) treatments on blueberry fruit texture and weight during postharvest storage in ‘Miss Lilly’ in 2019, and in ‘Premier’ and ‘Powderblue’ in 2019 and 2020

	Compression (kgF)			Puncture (kgF)			Fruit weight (g)		
Treatments/	PH^a^3	PH10	PH21	PH3	PH10	PH21	PH3	PH10	PH21
PH storage									
**‘Miss Lilly’ 2019**									
Control	0.26	0.26	0.23	0.17	0.17	0.16	1.31	1.34	1.32
Ethephon	0.27	0.28	0.25	0.18	0.18	0.18	1.2	1.21	1.24
ACC	0.43	0.27	0.23	0.16	0.17	0.15	1.18	1.22	1.13
*P*-value	ns	ns	ns	ns	ns	ns	ns	ns	ns
**‘Premier’ 2019**									
Control	0.21	0.21	0.18	0.14	0.14	0.13	1.19	1.22	1.19
Ethephon	0.21	0.19	0.18	0.13	0.12	0.12	1.36	1.17	1.22
ACC	0.19	0.17	0.16	0.13	0.13	0.12	1.31	1.31	1.16
*P*-value	ns	ns	ns	ns	ns	ns	ns	ns	ns
**‘Powderblue’ 2019**									
Control	0.21	0.19	0.16	0.15	0.14	0.14 b	1.16 a	1.21 a	1.19 a
Ethephon	0.21	0.20	0.19	0.16	0.16	0.16 a	0.99 b	1.08 b	0.99 b
ACC	0.22	0.22	0.19	0.16	0.16	0.16 a	0.99 b	0.94 c	0.96 b
*P*-value	ns	ns	ns	ns	ns	0.020	0.005	< 0.001	0.004
**‘Premier’ 2020**									
Control	0.24	0.25	0.2	0.19	0.22	0.21	1.87 a	1.66 a	1.74 a
Ethephon	0.26	0.27	0.25	0.22	0.24	0.22	1.41 b	1.33 b	1.31 b
ACC	0.24	0.25	0.22	0.19	0.19	0.2	1.74 a	1.63 a	1.63 a
*P*-value	ns	ns	ns	ns	ns	ns	< 0.001	0.050	0.008
**‘Powderblue’ 2020**									
Control	0.23	0.21	0.21 b	0.24 c	0.24	0.25	1.02 a	0.97	1
Ethephon	0.24	0.23	0.25 a	0.28 a	0.28	0.28	0.90 b	0.8	0.84
ACC	0.22	0.21	0.22 b	0.26 b	0.27	0.27	0.94 ab	0.85	0.9
*P*-value	ns	ns	0.022	0.005	ns	ns	0.047	ns	ns

^a^ postharvest (PH) storage at 3 days (PH3), 7 days (PH7) and 21 days (PH21)

Means followed by the same letter within a column and a given time-point after storage within a cultivar are not significantly different, according to ANOVA and Fischer’s LSD (*α* = 0.05)

**Table 2:** Effects of ethephon and 1-aminocyclopropane 1-carboxylic acid (ACC) treatments on titratable acidity (TA), total soluble solids content (TSS) and defect (%) during postharvest storage of blueberry fruit in ‘Miss Lilly’ in 2019, and in ‘Premier’ and ‘Powderblue’ in 2019 and 2020

	TA (% CA)			TSS (% Brix)			Defect (%)		
Treatments/	PH^a^3	PH10	PH21	PH3	PH10	PH21	PH3	PH10	PH21
PH storage									
**‘Miss Lilly’ 2019**									
Control	0.52	0.42	0.36	11.2	11.7	11.8	3.8	10.0	20.0
Ethephon	0.53	0.47	0.33	10.9	11.9	11.3	2.5	6.3	17.5
ACC	0.53	0.45	0.35	11.4	11.9	12.0	5.0	10.0	31.3
*P*-value	ns	ns	ns	ns	ns	ns	ns	ns	ns
**‘Premier’ 2019**									
Control	0.40	0.41 a	0.34	15.3 a	14.7 a	14.6	1.3	8.8	21.3
Ethephon	0.42	0.36 b	0.31	14.6 ab	14.4 a	14.4	1.3	6.3	13.8
ACC	0.39	0.35 b	0.32	13.4 b	13.2 b	13.1	2.5	11.3	20.2
*P*-value	ns	0.033	ns	0.020	< 0.001	ns	ns	ns	ns
**‘Powderblue’ 2019**									
Control	0.43 b	0.43 b	0.42	16.7 a	16.6 a	16.0	1.3	0	6.3
Ethephon	0.52 a	0.44 b	0.47	14.4 b	14.1 b	14.2	1.3	0	6.3
ACC	0.51 a	0.50 a	0.49	14.8 b	14.3 b	14.5	2.5	1.3	3.8
*P*-value	0.024	0.026	ns	0.002	0.004	ns	ns	ns	ns
**‘Premier’ 2020**									
Control	0.48	0.43	0.35	13.5	12.7	12.3	5.0	6.3	20.0
Ethephon	0.51	0.47	0.39	12.3	12.2	11.9	3.8	7.5	13.8
ACC	0.48	0.4	0.34	13.0	12.6	12.2	3.8	13.8	18.8
*P*-value	ns	ns	ns	ns	ns	ns	ns	ns	ns
**‘Powderblue’ 2020**									
Control	0.51	0.49	0.48	17.2 a	16.3	16.7	0	3.8	12.5 a
Ethephon	0.58	0.59	0.55	15.8 b	15.1	15.0	1.3	0	3.8 b
ACC	0.55	0.53	0.53	15.2 b	15.9	15.1	2.5	1.3	8.8 a
*P*-value	ns	ns	ns	0.005	ns	ns	ns	ns	0.012

^a^ postharvest (PH) storage at 3 days (PH3), 7 days (PH7) and 21 days (PH21)

Means followed by the same letter within a column and a given time-point after storage within a cultivar are not significantly different, according to ANOVA and Fischer’s LSD (*α* = 0.05)

Fruit weight was not different in ‘Miss Lilly’ and ‘Premier’ in 2019 in response to either PGR treatments (Table 1). In ‘Powderblue’ in 2019, ethephon and ACC treatments decreased fruit weight at PH3, PH10, and PH21 by 1.1 to 1.3-fold (Table 1). In 2020, ethephon treatments decreased fruit weight by 1.1 to 1.3-fold at all sampling dates in ‘Premier’ and at PH3 in ‘Powderblue’ (Table 1). However, in 2020, no fruit weight differences were noted between ACC and control treatments in any of the cultivars (Table 1).

Overall, ethephon and ACC treatments showed inconsistent effects on titratable acidity (TA) and total soluble solids (TSS) content across time-points, cultivars and between years (Table 2). In ‘Miss Lilly’ in 2019, no treatment effects on TA and TSS content were noted. In ‘Premier’ 2019, control treatment had higher TA and TSS content at PH10. In ‘Powderblue’ 2019, both ethephon and ACC treatments had higher TA at PH3, and lower TSS content at PH3 and PH10 compared to the control (Table 2). In 2020, TSS content was higher in control fruit only in ‘Powderblue’ and at PH3. The percentage of defective fruit was not affected by ethephon and ACC treatments, except in ‘Powderblue’ 2020, where ethephon treatment showed a decrease by around 9% compared to the control at PH21 (Table 2).

**Table 3:** Sample collection and measurement dates for determining the effects of ethephon and 1-aminocyclopropane 1-carboxylic acid (ACC) on fruit metabolism and ripening

	2019	2019	2019	2020	2020
Measurements/ Cultivar	‘Ms. Lilly’	‘Premier’	‘Powderblue’	‘Premier’	‘Powderblue’
Treatment date	1-May	14-Jun	21-Jun	7-Jun	26-Jun
Ripening rate (DAT)	0, 2, 5, 9	0, 3, 5, 7	0, 3, 5, 10	0, 3, 5, 7, 10	0, 3, 5, 7, 10
Ethylene (DAT)	2, 5	2, 4	2, 4	3, 5	1, 3, 5
Carbon dioxide (DAT)				3, 5	3, 5
Metabolite (DAT)				0, 3, 5, 10^#^	0, 3, 5, 10^#^
Gene expression (DAT)^*^				0, 1, 2, 3	0, 1, 2, 3
Harvest PH^a^ (DAT)	9^#^	7^#^	10^#^	10^#^	10^#^
PH analyses (PH)	3, 10, 21	3, 10, 21	3, 10, 21	3, 10, 21	3, 10, 21

^#^ Ripe fruit harvested, for all other time-points fruits were collected randomly

^*^ Only ethephon treatment compared to control

^a^PH: Postharvest

## Discussion

The current study indicated that ethylene-releasing PGRs increased the rate of ripening by enhancing the percentage of pink and ripe (blue) fruit, while reducing green fruit in one southern highbush and two rabbiteye blueberry cultivars. This is consistent with our previous findings and other studies which have shown that ethephon accelerates ripening in rabbiteye and northern highbush blueberry [10, 23, 24]. In this study, in comparison to ethephon, the rate of ripening following ACC application was in general slightly protracted (Fig. 1). Only in 2019, in ‘Premier’ the effect of PGRs in enhancing ripening was not evident. This was very likely due to advanced maturity of the fruit at the time of PGR applications. Both PGRs increased ethylene immediately after application (Fig. 2). However, this effect was transient as ethylene levels declined later during fruit development. Ethephon applications resulted in an immediate burst in ethylene production especially within 1 DAT (monitored in ‘Powderblue’ in 2020), whereas ethylene production peaked within 2–3 days after ACC applications. Ethephon releases ethylene non-enzymatically when the pH is greater than 3.5, usually following absorption by plant cells, and higher temperatures increase the rate of ethylene evolution [25, 26]. ACC conversion to ethylene occurs *via* ACC oxidase which is not rate-limiting during blueberry fruit ripening [9]. The faster rate of ethylene production following ethephon application may be attributed to a combination of immediate non-enzymatic conversion and high summer temperatures. Thus, based on the observations from this study ethylene release from ethephon application compared to that from ACC may have resulted in the accelerated ripening rates.

Previously, we demonstrated that the major sugars, Glc, Fru, and Suc accumulate continuously during blueberry fruit development extending well into the later stages of ripening [12]. During blueberry fruit ripening, Suc is broken down in the cytosol *via* SuSy and nINV, however much higher catabolism occurs in the vacuoles *via* vINV [12]. Sucrose catabolism *via* vINV and its breakdown to hexose sugars is important for sink activity and sustained carbon import into the fruit from source tissues [27, 28]. Therefore, in blueberries, higher expression of *vINV* may be associated with continuous import of sucrose into the fruit during ripening [12]. In this study an increase in the three major sugars was noted at 3 DAT in ‘Premier’ in response to either PGR treatments (Fig. 4A-C). This response was not observed in ‘Powderblue’ (Fig. 4D-F). Given the immediate and transient nature of metabolite alterations, it is plausible that in ‘Powderblue’, modifications in sugar concentrations occurred earlier, potentially prior to 3 DAT. Notably, *SuSy2* displayed increased expression after ethephon application only in ‘Powderblue’ at 2 DAT, and *vINV* at 2 DAT in ‘Premier’ and at 1 and 3 DAT in ‘Powderblue’. The transient increase in transcript abundance in both cultivars is indicative of an early response to ethylene-induced stimulation of sugar-metabolism related genes, especially that of *vINV*.

In climacteric fruits such as tomato, apple, and banana, starch reserves generated during fruit development are metabolized into sugars during fruit ripening with ethylene promoting this process [5, 29, 30]. Ethylene treatment of transgenic apples silenced for ethylene biosynthesis genes, stimulates starch hydrolysis, and increases Suc levels at 14 days after storage compared to control fruit [31]. In apple, pre-harvest application of ethephon increases starch degradation until 180 days, and the content of Glc, Fru, Suc until 60 days of PH storage [30]. The transcript abundance of multiple genes and/or enzyme activities associated with carbohydrate metabolism including AMYLASE, sucrose phosphate synthase (SPS), SuSy, vINV, nINV, and cwINV are enhanced, between 60 and 150 days after cold storage [30]. The ethylene signaling protein MaEIL2 binds to the promoters of genes coding for enzymes involved in starch degradation, positively regulating their expression, indicating a direct role for ethylene in starch metabolism and increasing soluble sugar accumulation during banana fruit ripening [32]. This is also supported by tomato ripening mutants such as *rin*, *nor*, and *Nr* which display inhibited climacteric ethylene production and a reduction in the abundance of Suc, Glc and Fru [33–35].

In non-climacteric fruits, that do not accumulate substantial amounts of starch, such as grapes, ethephon application at the rate of 500 mg·L^− 1^ one week before veraison, increases Glc, Fru and Suc for up to 15 days after treatment [36]. Ethylene promotes the expression of Suc catabolism-related genes, *cwINV*, *nINV*, and *SuSy*, observed at 5 and 10 days after treatment [36]. Application of ethylene to detached strawberry fruit at the white stage showed mixed results with one study suggesting it to be insensitive to this hormone [37] and other studies showing increased sugar concentration at 2 days after treatment [38, 39]. A comparison between climacteric plum and its non-climacteric bud mutant showed that ethylene stimulated sugar metabolism [40]. Through these studies it may be speculated that in climacteric fruits ethylene may primarily drive starch degradation thereby promoting sugar metabolism, whereas in non-climacteric fruits ethylene may stimulate sugar translocation and subsequent metabolism.

In fruit exhibiting atypical ripening behavior like kiwifruit, the initial fruit ripening phase is sensitive to, but progresses before detectable ethylene accumulation [41]. Regardless, ethylene plays an important role in stimulating starch degradation and sugar metabolism by inducing the expression of *β-amylase* and *SuSy* genes [42, 43]. These studies highlight the role of ethylene in regulating sugar metabolism across fruit with varied ripening behavior. Blueberry fruit are classified into a distinct category exhibiting atypical ripening physiology with an increase in ethylene during ripening [9]. In the current study, the transient and immediate increase in sugars and expression of related metabolism genes indicate the important role of ethylene in regulating sugar metabolism as a part of the ripening initiation program in blueberry. However, blueberry fruit do not accumulate starch reserves during development and are therefore dependent on continuous Suc import from the source [12]. Whether stimulation of sugar metabolism during fruit ripening noted in this study is aided by ethylene induced sugar translocation mechanisms in blueberry requires further investigation.

The concentration of major acids such as malate and quinate decrease during blueberry ripening, whereas citrate concentration is generally low and does not display ripening-related alterations [12]. In the current study, the application of either PGR decreased malate at 3 DAT in ‘Premier’, while only ethephon reduced it in both cultivars at 5 DAT (Fig. 5A, C). In addition, both ethephon and ACC increased respiration within 3 DAT in ‘Premier’ (Fig. 3A). This is in contrast to our previous study where ethylene treatments did not influence fruit respiration, however in that study detached fruit were incubated with ethylene at specific ripening stages [9]. Data from this study, combined with the approximately 2-fold increase in respiration rate during ripening [9], strengthens the evidence that ethylene promotes blueberry ripening.

In general, studies in both climacteric and non-climacteric fruit support an ethylene-induced decline in organic acids during ripening. In tomato, ripening mutants such as *rin*, *nor*, and *Nr* exhibit a decrease and delay in the decline of malate accumulation during ripening [34, 35]. In apple fruit, silencing of ethylene biosynthesis genes, resulted in a reduction in the loss of citrate and malate. However, upon ethylene treatment a decline of these organic acids were noted [31]. In grape, ethephon treatments decreased tartarate and malate concentrations, and downregulated *PEPC* and *MDH*, suggesting a decrease in malate synthesis [36]. Although strawberries treated with ethephon at various fruit developmental stages did not display altered concentrations of organic acids in ripe fruit, this study did not evaluate the transient effect of this treatment [44]. Overall, these studies suggest ethylene induced stimulation of organic acid metabolism, which is consistent with our observations in blueberry.

In this study, ethylene increased the transcript abundance of *PEPCK* at 3 DAT in ‘Premier’ (Fig. 5G). Although not evident in this study multiple transcripts of *PEPCK* from an RNA-Seq analyses showed up to 1.8-fold increase in response to ethephon at 1 and 2 DAT in ‘Powderblue’ fruit [11], suggesting conversion of malate to PEP. One of the fates for PEP is synthesis of shikimate, which can in-turn be channeled towards anthocyanin production. In this study, ethephon stimulated an immediate and transient increase (1–3 days) in anthocyanin biosynthesis gene expression such as that of *CHS* and *UFGT* (Fig. 6G-N). Concomitantly, both the PGRs promoted anthocyanin accumulation within 3–5 DAT (Fig. 6A-F). In tomato, carotenoid accumulation during fruit ripening is regulated by ethylene-dependent and independent processes [35]. External applications of ethylene at the green or white stage stimulated anthocyanin production in strawberries [37, 38]. Commercially, ethephon is used as a PGR to promote color development in citrus, and grape, that accumulate carotenoids and anthocyanins, respectively [45–47]. The stimulation of anthocyanin production following the application of ethylene-related PGRs in this study, led to an increase in the percentage of blue (ripe) fruit, consistent with previous reports [10, 11].

Previously, we established that ethylene plays an important role in ripening initiation by downregulating photosynthesis-related genes and interacting with other phytohormones [11]. In this study, the rapid stimulation of sugar, acid and anthocyanin metabolism within 5 days of PGR treatment indicates that ethylene plays an important role in regulating the metabolic programs of the fruit. Collectively, these findings suggest that ethylene coordinates multiple physiological processes, thereby initiating ripening in blueberry. However, findings from the current study suggested that the metabolic alterations were not sustained when ripe fruit were harvested at 10 DAT, possibly because the increase in ethylene evolution following PGR treatments was transient. This argument is also supported by the findings that in blueberry, ethylene is regulated developmentally and is not autocatalytic [9].

In the current study, ethephon and ACC did not display any consistent effects on fruit compression, puncture, TA, TSS content, and visual quality across different postharvest storage time points, among cultivars, or between years. These results are similar to those noted in our previous study with pre-harvest ethephon applications [10]. Previously, we noted that ethephon treatments showed a trend towards a slight decrease in fruit weight, although this was not significant [10]. In this study, fruit weight decreased significantly in response to either PGR treatments in ‘Powderblue’ in 2019 (by 11–22%) and after ethephon treatment for both cultivars in 2020 (by < 25%). These results suggest that accelerated developmental transition into ripening due to ethylene may limit fruit expansion that occurs during later stages of development, leading to a decrease in fruit weight.

## Conclusions

Overall, this study demonstrates that ethephon and ACC were equally effective in promoting ripening, although ACC displayed slightly protracted ripening rate compared to ethephon. The PGR treatments stimulated sugar and anthocyanin metabolism and decreased malic acid concentrations within 3–5 DAT compared to the control, and related gene expression within 1–3 DAT. Together with previous work, these data indicate that increased ethylene production during blueberry ripening facilitates developmental transition to ripening. No consistent or substantial effects of PGRs were noted on fruit quality attributes during postharvest storage, with the exception of a significant but inconsistent decrease in fruit weight. Blueberry fruit exhibit non-uniform ripening and are harvested multiple times which can increase harvesting cost and challenges with scheduling labor. Our study indicated that ethephon and ACC can promote ripening, and that these PGRs have the potential to be used as harvest tools to decrease the number of harvests by concentrating ripening. However, to use these PGRs as ripening aids, without any negative influence in fruit attributes it would be useful to identify lower application rates and evaluate their effects on ripening and fruit quality.

## Materials and methods

### Plant material

This study was performed with two rabbiteye (*Vaccinium ashei*) cultivars, Premier and Powderblue, in 2019 and 2020, and one southern highbush (*Vaccinium corymbosum* hybrids) cultivar, Miss Lilly, in 2019. Fruit were harvested from the cultivars, Premier and Powderblue, from the Durham Horticulture Farm, University of Georgia, Athens, GA, and Miss Lilly from the Alapaha Blueberry Research Farm, Alapaha, GA. The experimental design was completely randomized with four replications with one plant per experimental unit.

### Plant growth regulator treatments

Ethephon (Bayer CropScience, MO) and ACC (Valent Bioscience LLC, IL) were applied at 250 mg·L^− 1^ with 0.15% Latron-1956 (Southern Agricultural Insecticides, Inc., NC) as a surfactant. Control plants were treated with only 0.15% Latron-1956. All PGR applications were performed using a hand pump sprayer on the whole plant until runoff in the morning (before 10:00 AM). Approximately 15–20% of fruit were ripe at the time of PGR application, with the exception in 2019 in ‘Premier’, where 55–60% of fruit were ripe. Prior to PGR applications, fruit from S1, S2, and S3 stages of early fruit development, and fully ripe fruit (S8 stage) [48] were removed from the tagged branches to maintain developmental uniformity during sample collection. For each plant, shoots containing 50–150 fruit were tagged to determine the rate of ripening (3 shoots), ethylene evolution (2–3 shoots), carbon dioxide measurement (2 shoots), and metabolite analysis (3 shoots). The treatment application dates and collection of fruit for various measurements are summarized in Table 3 and described below.

### Rate of ripening

Green, pink, and ripe fruit were counted from the tagged branches. For green fruit, all fruit that were not at the pink and ripe stages were counted, with fruit mostly from S4-S6 stages [12, 48]. Pink fruit (S7 stage) displayed 50 to 100% pink peel coloration and in ripe fruit (S8 stage) the peel was completely blue. Finally, the percentage of fruit categorized into green, pink, and ripe were calculated to determine the ripening rate among treatment and control groups. An exception occurred in ‘Premier’ in 2019, due to fruit being at an advanced growth stage before treatment application. In this case, green and pink stage fruit were grouped together for counting. The rate of ripening was determined at regular intervals after PGR treatments (Table 3). In 2019, fruit were counted at 0, 2, 5, and 9 days after treatment (DAT) in ‘Miss Lilly’, 0, 3, 5, and 7 DAT in ‘Premier’, and 0, 3, 5, and 10 DAT in ‘Powderblue’. In 2020, fruit were counted at 0, 3, 5, 7, and 10 DAT in both ‘Premier’ and ‘Powderblue’.

### Ethylene and carbon dioxide measurements

Fruit were sampled randomly from the tagged branches and represented a mixture of different developmental stages (green, pink, and ripe). Ethylene measurements were conducted in both 2019 and 2020, whereas carbon dioxide (CO_2_) measurements were taken only in 2020 (Table 3). In 2019, fruit were collected at 3 and 5 DAT for ‘Miss Lilly’. For ‘Premier’ and ‘Powderblue’, fruit were collected at 2 and 4 DAT in 2019, and 3 and 5 DAT in 2020. Ethylene and CO_2_ measurements were conducted on the same day after fruit collection for ‘Premier’ and ‘Powderblue’. Fruit from ‘Miss Lilly’ were collected in clamshells and transported the same day to Athens, GA where they were stored in a walk-in cooler set to 4 °C and 90–95% relative humidity (RH). The following day, fruit were placed in the laboratory bench for several hours to equilibrate to room temperature prior to ethylene and CO_2_ measurements.

To measure ethylene, approximately 25 g of fruit were placed in a 135 mL glass jar, which was then tightly sealed with a lid fitted with a septum. Fruit were incubated for four hours at room temperature, after which 1 mL of the headspace gas was taken from the jar using the syringe with a needle and injected into the Gas Chromatograph (GC) (GC-17 A, Shimadzu, MD) equipped with a Hayesep-N micro packed column (Restek, PA) and a flame ionization detector as described previously [9]. The sample chromatogram’s peak area was quantified using a standard curve from ethylene standards prepared in the laboratory. The ethylene production rate was expressed as nL·g^− 1^ ·h^− 1^. For CO_2_ measurements, approximately 10 g of fruit were placed into a 495 mL glass jar and tightly capped with a lid fitted with the septum. Fruit were incubated for 1 h at room temperature. Then, 60 mL of the headspace gas was taken from the jar using the syringe with a needle and injected into the CO_2_ analyzer (Model 902, Quantek, MA). The CO_2_ production rate was expressed as µL. g ^− 1^.h ^− 1^.

### Metabolite analysis

The effect of ethephon and ACC on sugar, acid, and anthocyanin-related metabolites was evaluated at 3 and 5 DAT (early response) and at 10 DAT (sustained response) (Table 3). Fruit from the tagged branches were collected at early time points and consisted of random pools of different developmental stages at 0, 3 and 5 DAT in 2020 from ‘Premier’ and ‘Powderblue’. To evaluate long-term response, only ripe stage fruit were collected at 10 DAT. Fruit were immediately frozen in liquid N_2_ and stored at -80 °C until analysis. Sugars, acids, and amino acids were measured using a gas chromatograph equipped with a flame ionization detector (GC-FID) (GC-2014; Shimadzu, Japan). The details of methods used for metabolite analysis are described in Acharya et al. (2024) [12]. For every metabolite, a standard curve was generated for quantification using commercially available standards. Anthocyanins were determined using a high-performance liquid chromatography (HPLC) equipped with a photodiode array detector (PAD) (Waters, MA). All anthocyanin compounds were quantified using the standard curve prepared from malvidin-3-O galactoside (Mal 3-gal).

### Quantitative RT-PCR

Transcript abundance of sugar, acid, and anthocyanin metabolism-related genes were determined by quantitative real-time polymerase chain reaction (qRT-PCR). Gene expression analyses were performed using fruit treated with ethephon and control from 2020. As changes in gene expression are expected to precede those in metabolite accumulation, a random pool of fruit samples from ‘Premier’ and ‘Powderblue’ were obtained at 0, 1, 2, and 3 DAT (Table 3). Fruit samples at 0 and 3 DAT were the same as those used to determine metabolites. Fruit samples were collected, stored at -80 °C, and finely ground in liquid N_2_ for RNA extraction. The modified cetyltrimethylammonium bromide (CTAB) method used for the total RNA extraction described previously [49] was adapted for his study. After treatment of 1 µg of RNA with DNase, complementary DNA (cDNA) was prepared using reverse transcriptase (Promega, WI). The qRT-PCR reaction was set-up using the PowerUp SYBR Green master mix reagent (ThermoFisher Scientific, MA) and a MX3005P Real-Time PCR system (Agilent, CA). Genes related to sugar, acid and anthocyanin metabolism were shortlisted based on previous studies [11, 12] and their corresponding primer sequences are presented in Table S3 [11, 12]. Gene normalization, relative transcript abundance, and data analysis was performed as described previously [12]. The transcript abundance of a gene in response to PGR treatments are presented as fold change relative to the control treatment at 0 DAT.

### Postharvest measurement

Fully ripe fruit were harvested at 9, 7, and 10 DAT in ‘Miss Lilly’, ‘Premier’, and ‘Powderblue’, respectively, in 2019, and at 10 DAT in ‘Premier’ and ‘Powderblue’ in 2020 (Table 3). Fruit were stored in clamshells in a walk-in cooler maintained at 4 °C and 90–95% RH. Fruit quality attributes were determined at three-time points during postharvest (PH) storage, i.e., 3 days after PH storage (PH3), 10 days after storage (PH10), and 21 days after storage (PH21). The percentage of healthy fruit, texture (compression and puncture), weight, total soluble solid (TSS) content, and titratable acidity (TA) were evaluated during the PH stages as described previously [10].

### Statistical analysis

Data analysis and visualization were performed using the R-studio 2023 (R core 2023, Vienna, Austria). The treatment effects were analyzed using ANOVA and Fisher’s least significant difference (LSD) at the 0.05 (*α*) level of significance. Only statistically significant effects due to PGR treatments are explained in the Results section.

## Electronic supplementary material

Below is the link to the electronic supplementary material.

Supplementary Material 1

Supplementary Material 2

Supplementary Material 3

## Data Availability

Data is available as figures, tables and additional files provided in the manuscript. Any additional data will be provided upon request to the corresponding author.

## Abbreviations

ACC: 1-aminocyclopropane 1-carboxylic acid
CHS: CHALCONE SYNTHASE
Cya 3-ara: Cyanidin 3-arabinoside
Cya 3-gal: Cyanidin 3-galactoside
Cya 3-glu: Cyanidin 3-glucoside
DAT: Days after treatment
Del 3-ara: Delphinidin 3-arabinoside
Del 3-gal: Delphinidin 3-galactoside
Del 3-glu: Delphinidin 3-glucoside
Fru: Fructose
Glc: Glucose
INV: Invertases
Mal 3-ara: Malvidin 3-arabinoside
Mal 3-gal: Malvidin 3-galactoside
Mal 3-glu: Malvidin 3-glucoside
MDH: Malate dehydrogenase
mETC: Mitochondrial electron transport chain
OAA: Oxaloacetate
Peo 3-ara: Peonidin 3-arabinoside
Peo 3-gal: Peonidin 3-galactoside
Peo 3-glu: Peonidin 3-glucoside
PEP: Phophoenolpyruvate
PEPC: Phophoenolpyruvate carboxylase
PEPCK: Phophoenolpyruvate carboxykinase
Pet 3-ara: Petunidin 3-arabinoside
Pet 3-gal: Petunidin 3-galactoside
Pet 3-glu: Petunidin 3-glucoside
PFK: PHOSPHOFUCTOKINASE
PGR: Plant growth regulators
PH: Postharvest
PK: PYRUVATE KINASE
SPS: Sucrose phosphate synthase
Suc: Sucrose
SuSy: Sucrose synthase
TA: Titratable acidity
TSS: Total soluble solids
UFGT: UDP-GLUCOSE FLAVONOID 3-O-GLUCOSYLTRANSFERASE
