# Supplementary material for: Ethylene-releasing plant growth regulators promote ripening initiation by stimulating sugar, acid and anthocyanin metabolism in blueberry (Vaccinium ashei)
Source: BMC Plant Biol. 2025 Jun 5;25:766. doi: 10.1186/s12870-025-06799-x (PMC12139259; doi:10.1186/s12870-025-06799-x)
Supplement: Supplementary file 1 — Supplementary Material 1 [file 12870_2025_6799_MOESM1_ESM.docx]

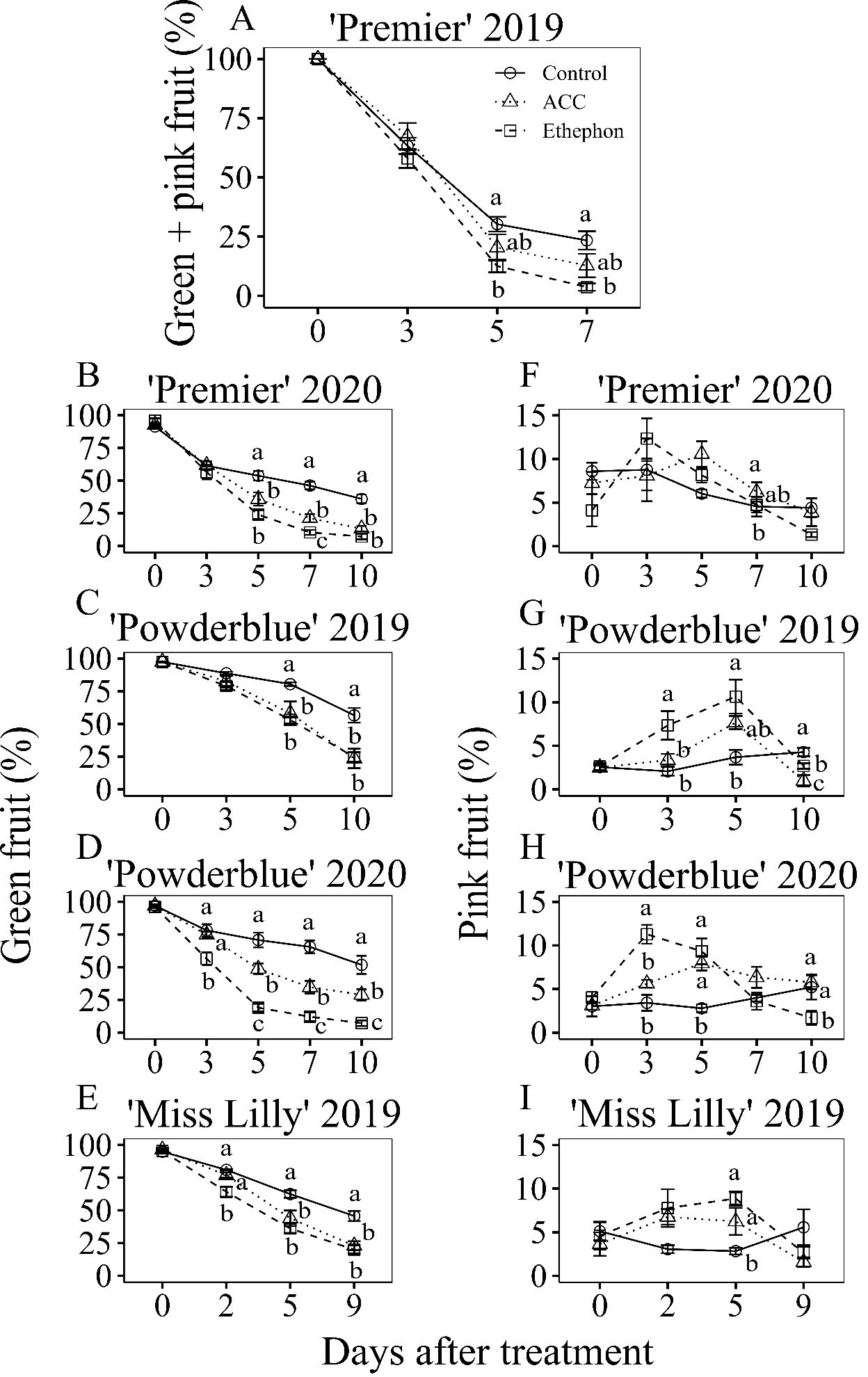


**Supplementary Material 1: Figure S1**: **Effects of ethephon and 1-aminocyclopropane 1-carboxylic acid** (**ACC) treatments on the rate of ripening in blueberry fruit.** Percentage of unripe fruit (green and pink fruit, A), green (B, C, D, E) and pink (F, G, H, I) fruit in control and in response to 1-aminocyclopropane 1-carboxylic acid (ACC) and ethephon treatments in three cultivars, Premier (A, B, F), Powderblue (C, D, G, H ) , and Miss Lilly (E, I), in 2019 (A, C, E, G, I) and 2020 (B, D, F, H). Mean ± S.E. (*n* = 4) are presented. Statistical analyses were performed using ANOVA followed by Fischer’s Least Significant Difference (LSD; *α* = 0.05). The same letter above the symbols indicates no statistically significant differences across treatments within a given day after treatment.
