# Supplementary material for: Ethylene-releasing plant growth regulators promote ripening initiation by stimulating sugar, acid and anthocyanin metabolism in blueberry (Vaccinium ashei)
Source: BMC Plant Biol. 2025 Jun 5;25:766. doi: 10.1186/s12870-025-06799-x (PMC12139259; doi:10.1186/s12870-025-06799-x)
Supplement: Supplementary file 2 — Supplementary Material 2 [file 12870_2025_6799_MOESM2_ESM.docx]

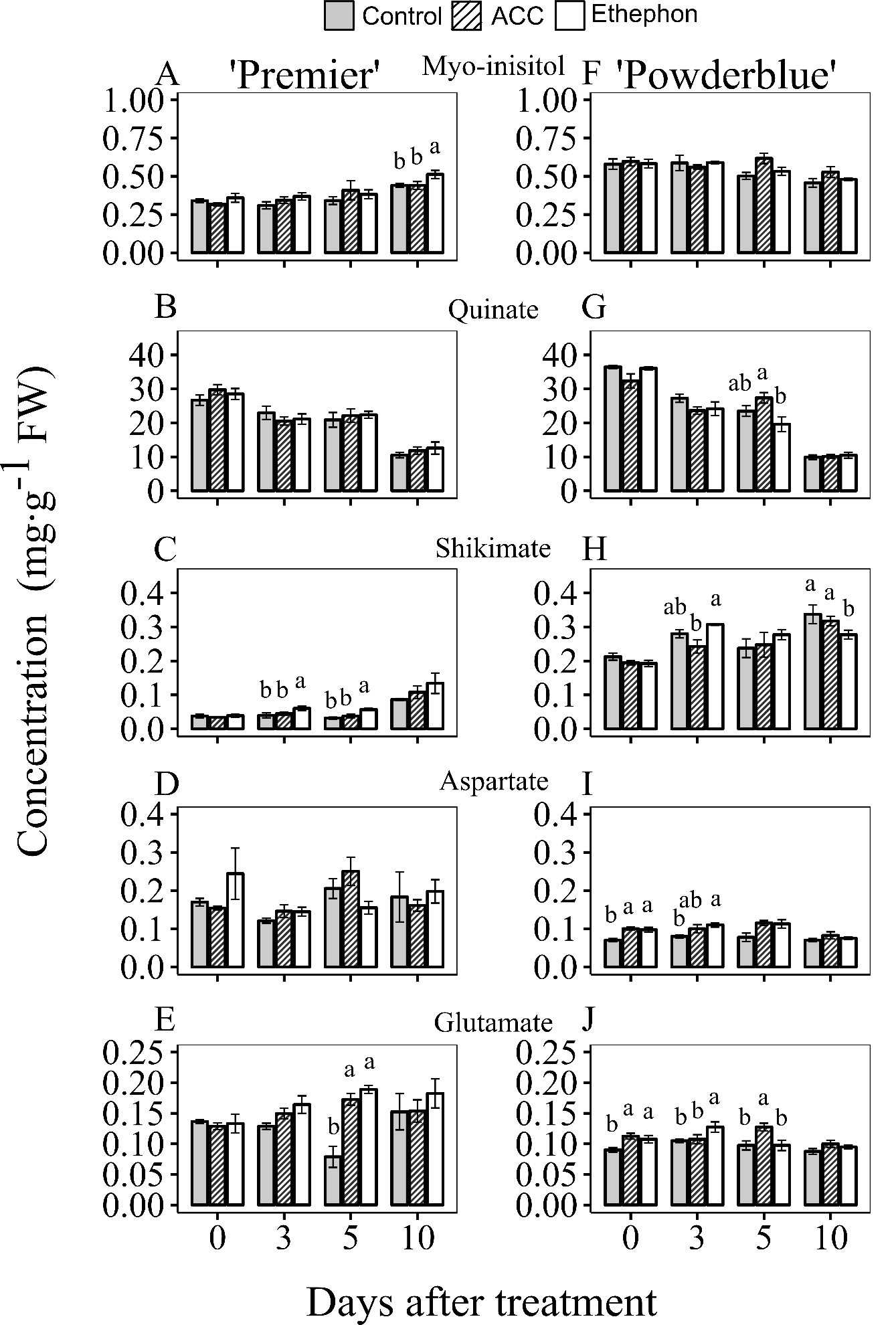


**Supplementary Material 2: Figure S2**: **Effects of ethephon and 1-aminocyclopropane 1-carboxylic acid** (**ACC) treatments on blueberry fruit metabolism.** Concentrations of myo-inisitol (A, F), quinate (B, G), shikimate (C, H), aspartate (D, I), and glutamate (E, J) in control fruit and after treatments with ethephon and ACC in ‘Premier’ (A-E) and ‘Powderblue’ (F-J) in 2020 at 0, 3, 5 and 10 d after treatment (DAT). A pool of fruit was randomly collected from tagged branches. At 10 DAT, only ripe fruit were harvested. Mean ± S.E. (*n* = 4) are presented. Statistical analysis were performed using ANOVA followed by Fischer’s Least Significant Difference (LSD; *α* = 0.05). The same letter above the bars indicates no statistically significant differences across treatments within a given day after treatment.
